# Targeting MT1-MMP as an ImmunoPET-Based Strategy for Imaging Gliomas

**DOI:** 10.1371/journal.pone.0158634

**Published:** 2016-07-27

**Authors:** A. G. de Lucas, A. J. Schuhmacher, M. Oteo, E. Romero, J. A. Cámara, A. de Martino, A. G. Arroyo, M. Á. Morcillo, M. Squatrito, J. L. Martinez-Torrecuadrada, F. Mulero

**Affiliations:** 1 Biomedical Application of Radioisotopes Unit, Centro de Investigaciones Energéticas, Medioambientales y Tecnológicas (CIEMAT), Madrid, Spain; 2 Seve Ballesteros Foundation Brain Tumour Group, Spanish National Cancer Research Centre (CNIO), Madrid, Spain; 3 Histopathology Unit, Spanish National Cancer Research Centre (CNIO), Madrid, Spain; 4 Matrix Metalloproteases Lab, Spanish National Center for Cardiovascular Research (CNIC), Madrid Spain; 5 Proteomics Unit, Spanish National Cancer Research Centre (CNIO), Madrid, Spain; 6 Molecular Imaging Unit, Spanish National Cancer Research Centre (CNIO), Madrid, Spain; Swedish Neuroscience Institute, UNITED STATES

## Abstract

**Background:**

A critical challenge in the management of Glioblastoma Multiforme (GBM) tumors is the accurate diagnosis and assessment of tumor progression in a noninvasive manner. We have identified Membrane-type 1 matrix metalloproteinase (MT1-MMP) as an attractive biomarker for GBM imaging since this protein is actively involved in tumor growth and progression, correlates with tumor grade and is closely associated with poor prognosis in GBM patients. Here, we report the development of an immunoPET tracer for effective detection of MT1-MMP in GBM models.

**Methods:**

An anti-human MT1-MMP monoclonal antibody (mAb), LEM2/15, was conjugated to p-isothiocyanatobenzyl-desferrioxamine (DFO-NCS) for ^89^Zr labeling. Biodistribution and PET imaging studies were performed in xenograft mice bearing human GBM cells (U251) expressing MT1-MMP and non-expressing breast carcinoma cells (MCF-7) as negative control. Two orthotopic brain GBM models, patient-derived neurospheres (TS543) and U251 cells, with different degrees of blood-brain barrier (BBB) disruption were also used for PET imaging experiments.

**Results:**

^89^Zr labeling of DFO-LEM2/15 was achieved with high yield (>90%) and specific activity (78.5 MBq/mg). Biodistribution experiments indicated that ^89^Zr-DFO-LEM2/15 showed excellent potential as a radiotracer for detection of MT1-MMP positive GBM tumors. PET imaging also indicated a specific and prominent ^89^Zr-DFO-LEM2/15 uptake in MT1-MMP+ U251 GBM tumors compared to MT1-MMP- MCF-7 breast tumors. Results obtained in orthotopic brain GBM models revealed a high dependence of a disrupted BBB for tracer penetrance into tumors. ^89^Zr-DFO-LEM2/15 showed much higher accumulation in TS543 tumors with a highly disrupted BBB than in U251 orthotopic model in which the BBB permeability was only partially increased. Histological analysis confirmed the specificity of the immunoconjugate in all GBM models.

**Conclusion:**

A new anti MT1-MMP-mAb tracer, ^89^Zr-DFO-LEM2/15, was synthesized efficiently. In vivo validation showed high-specific-contrast imaging of MT1-MMP positive GBM tumors and provided strong evidence for utility of MT1-MMP-targeted immunoPET as an alternate to nonspecific imaging of GBM.

## Introduction

Glioblastoma Multiforme (GBM) is the most common malignant tumor of the central nervous system (CNS) in adults. It belongs to a larger class of tumors known as glioma which arise from the astrocytic glial cells[1]. The World Health Organization has divided astrocytic-derived tumors into four grades based on their ability to infiltrate the surrounding brain tissue. Grade I glioma consists of benign pilocytic and other non-infiltrating tumors, while Grade II, III, and IV comprise infiltrating gliomas with increased malignancy[2]. GBM is a Grade IV glioma, corresponding with the most malignant form and has the worst prognosis. These tumors are also the most treatment-resistant and are difficult to image because of their diffuse infiltrative and proliferative features. Despite the recent advances in the understanding of their biology and in multimodality diagnostic and therapeutic procedures, these tumors maintain a high recurrence after treatment with very poor prognosis and increasing morbidity and mortality.

Clinical management is often compromised by an imprecise delineation of tumor boundaries, lack of assessment of the tumor sensitivity to a given therapy and late detection of recurrences. The currently employed imaging diagnostic tools, computed tomography (CT) and magnetic resonance imaging (MRI) provide excellent anatomical information on the localization of brain lesions, but are frequently not able to differentiate tumor tissue from other concurrent processes such as inflammation, edema or bleeding that give rise to under or over estimation of the actual extension of the tumor. In addition, the tumor response to treatment is conventionally measured by size change with MRI; however, this method does not consider the biological steps preceding the tumor reduction and prognostic evaluation cannot be obtained until weeks after the treatment initiation. These drawbacks make necessary to seek other non-invasive molecular imaging-based technologies that allow efficient and safe diagnosis providing additional and complementary biological information of the tumor. Positron Emission Tomography (PET) is widely used in oncology for staging, monitoring the efficacy of a given treatment and follow-up of a tumor recurrence because it offers an in vivo quantitative and comprehensive evaluation at the molecular level[3]. Conventional ^18^F-Fluorodeoxyglucose (^18^F-FDG) imaging is of limited usefulness for imaging GBM due to high levels of glucose uptake by normal brain resulting in a low signal-background ratio. Additionally, increase in FDG uptake is not a cancer-specific process as it has also been observed in inflammatory lesions and may lead to false-positive results[4]. Consequently, several preclinical and clinical studies have investigated the use of alternative PET radiotracers, such as labeled amino acids and their analogs ^11^C-Methionine (^11^C-MET), ^18^F-fluoroethyl-tyrosine (^18^F-FET) and ^18^F-fluoro-dihydroxyphenylalanine (^18^F-FDOPA)[5–7]; nucleoside radiotracers such as ^18^F-Fluorothymidine (^18^F-FLT)[8,9] with specificity for thymidine kinase-1 (TK-1) whose activity is elevated in the normal cellular S-phase and neoplastic tissues[10] or hypoxic tracers as ^18^F-Fluoromisonidazole (^18^F-FMISO)[11]. Despite of showing a better detection of neoplastic tissue and treatment monitoring than glucose imaging, there is still a clinical need to analyze more specific molecular events and processes that could have a major impact on the understanding of glioma biology, for grading and prognosis and on selection and monitoring of therapies. A promising option to improve diagnostic imaging is the immunoPET, which combines the high sensitivity, and quantitative capabilities of PET with the specificity and selectivity of monoclonal antibodies (mAb) against a given tumor cell surface marker. This combination makes this technique comparable to conduct a noninvasive, in vivo quantifiable, three-dimensional full-body immunohistochemistry allowing the diagnosis and monitoring of patients over time in a non-invasive manner

Membrane type 1-Matrix Metalloproteinase (MT1-MMP, also known as MMP14) has emerged as an attractive biomarker for tumor-targeted antibody development since this protein is crucial for the progression, invasion, migration and angiogenesis of tumors[12,13]; and specifically, its expression correlates with tumor grade and it is associated with reduced survival in gliomas[14,15] and other cancers[16]. In addition, a growing body of evidence reveals that overexpression of MT1-MMP plays also a significant role in promoting gliomagenesis[17]. MT1-MMP is broadly expressed in a variety of tissues and cell types including endothelial cells [18]. It belongs to a subset of zinc-dependent membrane-anchored MMPs able to degrade the basement membrane and proteins of the extracellular matrix (ECM)[19], cell adhesion molecules, cytokines, growth factors and receptors[20]. Many studies have highlighted its potential also as a therapeutic target in glioblastoma and other cancers[21,22]. In fact, a small molecule inhibitor, Marimastat, targeting MMP active site was tested in clinical trials with GBM patients[23,24]. However, this inhibitor failed because of unspecific inhibition of other MMPs with structurally conserved active sites leading to undesired side effects. Nonetheless, several highly-specific inhibiting antibodies such as DX-2400[25], 9E8[26] or LEM-2/15[27,28] have recently revived MT1-MMP as promising therapeutic target[29]. Besides, MT1-MMP would represent a candidate biomarker that can be leveraged for diagnostic purposes using specific probes, providing additional valuable information. Several studies using specific antibodies or peptides have demonstrated that molecules capable of tracing MT1-MMP might be used as imaging agents in several cancer models[30–32].

In the present study, we report the successful noninvasive detection of GBM tumors by PET imaging, using a radiolabeled MT1-MMP-specific mAb (LEM2/15) in mice xenografted with a glioma cell line expressing MT1-MMP or with patient-derived orthotopically implanted glioma tumor neurospheres. As a PET nuclide, we used ^89^Zr with a half-life (t_1/2_) of 78.4 h that is compatible to pharmacokinetics of intact antibodies (typically 2–4 days). The anti-MT1-MMP antibody was functionalized with an isothiocyanate-bearing derivate of desferrioxamine (DFO-NCS) as the ^89^Zr chelator. Our results support the feasibility of using noninvasive antibody-mediated PET imaging based on MT1-MMP marker to specifically visualize GBM tumors for diagnostic purposes.

## Materials and Methods

### Cell lines

GBM cell lines and neurospheres were kindly provided in 2012 by the Holland and Mellinghoff laboratories (MSKCC). SK-mel-103 cells were received from the Soengas laboratory (CNIO) in 2013. U251, T98G, U87-MG, SF-268, SF-295, MCF-7 and SK-mel103 cell lines were grown at 37°C in Dulbecco's Modified Eagle's Medium (Sigma) containing 10% FBS (Sigma). TS516, TS543, TS568, TS676 neurospheres are cultured in suspension in human NSC Basal Medium with NSC proliferation supplement, 1 mg/ml heparin (Stem Cell Technologies), 10 ng/ml recombinant human EGF (Invitrogen) and 20 ng/ml recombinant human basic FGF (Sigma). Primary tumor spheres were collected, mechanically disaggregated to a single-cell suspension and propagated by serial passaging.

### Protein isolation and immunoblotting

Whole protein lysates were isolated from cells lysed in NP-40 lysis buffer (0.5% NP-40, 50 mM Tris-HCl [pH 7.5], 50 mM NaCl, 1x phosphatase inhibitor cocktail set II (Calbiochem) and protease inhibitor cocktail set III EDTA-free (Calbiochem), and protein quantified by BCA assay (Pierce). Protein lysates were (100 μg per lane) separated by SDS-PAGE and transferred to nitrocellulose membranes (Amersham) for immunoblotting. Membranes were probed with antibodies against MT1-MMP (LEM2/15) and detected using HRP-conjugated anti-mouse antibodies (Dako) using chemiluminescence detection (ECL, Amersham).

### Flow cytometry

Cell lines were detached from dishes using Cell Dissociation Buffer, enzyme-free, PBS (Life Technologies). After counting, single cell suspensions in FACS buffer (1% IgG Free BSA in PBS (Sigma)) were incubated with 1μl of Fc Block (BD) for every million cells for at least 15 min at 4°C. Cells were then stained with the LEM 2/15 purified antibodies at the indicated concentrations for 15 min at 4°C, washed twice with FACS buffer. Cells were then incubated with appropriate fluorophore-conjugated secondary antibody (Molecular Probes, Invitrogen) at a dilution 1:500 for 15 min at 4°C, washed with FACS buffer, and resuspended in FACS buffer containing DAPI (5mg/ml diluted 1:5,000) for live/dead cell exclusion. Antibodies used for flow cytometry include LEM 2/15 obtained from ascytes, purified from cell culture, DFO conjugated and ^89^Zr labeled and decayed. For analysis, samples were run on a BD LSR II (Becton Dickinson), and all subsequent compensation and gating performed with FlowJo analysis software (TreeStar).

### Radiolabeling of antibody

Hybridoma cells of LEM2/15 were cultured as described previously[27], and the mAb was purified from the hybridoma supernatant according to standard methods using Protein A-based chromatography. The bifunctional chelator DFO-NCS (Macrocyclics, Dallas, TX) was conjugated to LEM 2/15 and subsequent ^89^Zr radiolabeling was performed by adaptation of published protocols[33,34]. ^89^Zr (*T*_1/2_ = 78.4 h, *β*^+^ = 22.6%; ~2.7 GBq/ml supplied in 1 M oxalic acid) was obtained from BV Cyclotron VU (Amsterdam, The Netherlands).

For conjugation, 2 mg of LEM 2/15 in 1 ml solution at pH 9.0, adjusted with 0.1 M Na_2_CO_3_ (max. 100 μl), were mixed with DFO-NCS (dissolved in DMSO at a concentration 3.5 mM) at a molar ratio of 1:5. The reaction was incubated for 40 min at 37°C. Non-conjugated chelator was removed by G25-Sephadex size-exclusion chromatography using a PD10 column (GE Healthcare Life Sciences) and 5 mg/ml gentisic acid in 0.25 M sodium acetate trihydrate (pH 5.4–5.6) as eluent.

For radiolabeling, the required volume of ^89^Zr-oxalic acid solution corresponding to 37–74 MBq was adjusted to a total volume of 200 μl using 1 M oxalic acid, 90 μl of 2 M Na_2_CO_3_ were added and incubated for 3 min at room temperature. One ml of 0.5 M HEPES and 710 μl of DFO-LEM 2/15 (1 mg/ml) were subsequently added and incubated at room temperature for 90 min on a rotating shaker. pH was checked to be at 7.0–7.5. Finally, the reaction mixture was loaded onto a previously equilibrated PD-10 column and eluted with phosphate-buffered saline (PBS) into fractions of 500 μL. After purification, the collected fractions were measured in a dose calibrator (IBC, Veenstra Instruments). Quality control was performed by instant thin-layer chromatography (ITLC) on ITLC strips (model 150–771, Biodex) using 0.02 M citrate buffer (pH 5.0):acetonitrile (9:1) as eluent.

The stability of ^89^Zr-DFO-LEM 2/15 was investigated by incubation in human serum for 7 days at 4°C and 37°C. The radiochemical purity was determined by ITLC as discussed above.

The DFO-NCS to LEM 2/15 molar ratio was determined before removing unconjugated chelate. Thus, a small aliquot of the reaction mixture was reacted with a tracer amount of ^89^Zr-oxalic acid for 90 min at room temperature. The mixture was put on a PD-10 column and eluted with PBS. The percentage of radioactivity present at the antibody fraction was then determined. The number of chelating molecules incorporated per antibody was calculated from the labeling efficiency and the molar ratio of added chelating agent to antibody.

The same procedure described for ^89^Zr-DFO-LEM 2/15 was followed to produce the ^89^Zr-labeled non-specific mouse IgG1 (BioXCell, USA) used as isotype control antibody (^89^Zr-DFO-IgG1).

### Subcutaneous and orthotopic tumor mouse models

For subcutaneous heterotopic xenografts 750,000 U251 or 1,000,000 MCF-7 cells were resuspended in 200μl of a 1:1 mix of DMEM (Sigma) with Matrigel (BD Biosciences). Next, the Matrigel:DMEM-cells mixture was injected subcutaneously into the flanks of 6 weeks athymic nude mice (Nude-Foxn1nu, Harlan Laboratories). Tumors were allowed to develop until palpable prior immunoPET analysis and were measured 3 times per week by caliper. Mice were sacrificed when the tumor mass reached a maximum size of 1500 mm^3^ or tumor ulceration was observed or mice were symptomatic from their tumors which included signs of lethargy, poor grooming, weight loss and hunching.

For orthotopic xenografts, U251 cells and primary TS543 neurospheres were transduced with a TK-GFP-Luciferase reporter plasmid (TGL) to monitor tumor growth by bioluminescence measurements and were described previously[35]. TS543 cells were previously used in intracranial PET imaging studies[36]. To develop intracranial tumors athymic nude mice (Harlan) were fully anesthetized with 10mg/ml ketamine and 1mg/ml xylazine and were subcutaneously injected with 50μl of the local anesthetic 0.25% bupivacaine at the surgical site. Mice were intracranially injected with 1.5μl containing 2,5 x 10^5^ U251-TGL cells or 5 x 10^4^ TS543-TGL (resuspended in PBS) at 6 weeks of age using a fixed stereotactic apparatus (Stoelting). Injections were made to the right frontal cortex, approximately 1 mm caudal and 1.5 mm lateral from bregma, and at a depth of 2 mm using a Hamilton syringe (Hamilton) as described previously[37]. Tumor growth was monitored by luminescence. Mice were injected with D-Luciferin (150 mg/kg). 15 minutes after injection, images were acquired for 5 seconds using an IVIS200 machine (Perkin Elmer). Bioluminescence analysis was performed using Living Image software, version 2.50. When tumors were in a positive growth phase determined by bioluminescence imaging (BLI) output, immunoPETs were performed. Mice were monitored by BLI every 3 days and sacrificed at the time points described or when they became symptomatic from their tumors, which included signs of lethargy, poor grooming, weight loss, hunching, macrocephaly or seizures. All animal experiments were performed according to protocols approved by the CNIO-ISCIII Ethics Committee for Research and Animal Welfare (CEIyBA) and they were performed in accordance with the guidelines stated in the International Guiding Principles for Biomedical Research Involving Animals, developed by the Council for International Organizations of Medical Sciences (CIOMS).

### Pharmacokinetic and biodistribution studies

Four groups of 4 female athymic mice each bearing U251 cells in flanks were injected i.v. (tail vein) with 1.5 MBq (20 μg) of ^89^Zr-DFO-LEM 2/15. Mice were euthanized at 1, 2, 4 and 7 days after injection by cervical dislocation under anesthesia with isofluorane in O_2_ and blood was immediately collected by cardiac puncture. For biodistribution studies organ tissues and tumors were excised, wet-weighed and counted for radioactivity with a gamma-counter (2470 Wizard^2^, PerkinElmer), along with a standard sample of the injected dose. Tissue activity was expressed as percentage injected dose per gram of tissue (%ID/g). Subsequently, tumors were formalin-fixed and stored at 4°C for immunohistochemical assays. For pharmacokinetic studies, blood samples were collected in heparinized tubes and centrifuged at 3000 rpm for 10 min to obtain plasma. Plasma concentrations of radioactivity were calculated as the percent injected dose per mL (%ID/mL) and were plotted versus the time post-injection. Pharmacokinetic parameters were estimated by noncompartmental analysis[38] using the computer program WinNonlin Professional, version 5.2 (Pharsight Corporation, Mountain View, CA, USA).

### microPET/CT imaging

Eleven mice bearing breast cancer cells (non-expressing MT1-MMP) in one flank and GBM cells (expressing MT1-MMP) in the contralateral side were injected i.v. (tail vein) with 1.5 MBq (20 μg) of ^89^Zr-DFO-LEM 2/15 and scanned with a small-animal Argus PET-CT scanner (SEDECAL, Madrid, Spain). The PET studies (energy window 250–700 KeV and 30 min static acquisition) and CT (voltage 45 kV, current 150 μA, 8 shots, 360 projections and standard resolution) were performed at various time points post-injection (1, 2, 4 and 7 days) in mice anesthetized by inhalation of 2–2.5% Isofluorane. The PET images were reconstructed using a 2D-OSEM (Ordered Subset Expectation Maximization) algorithm (16 subsets and two iterations), with random and scatter correction. Manually drawn regions of interest (ROIs) were used to determine the mean radiotracer accumulation in units of %ID/g tissue (decay corrected to the time of injection) in both tumors, brain (used as background tissue), and heart (used as blood pool). Images were analyzed using the image analysis software ITK-SNAP (www.itksnap.org). With the aim to obtain a more accurate quantification of the radiotracer in tumors, PET images were corrected for partial volume effect using tumor volume segmented from coregistered CT images. Recovery coefficients (RC, ratio between measured and true activity and used to correct the partial volume effect), were calculated using the phantom described in the NEMA NU 4–2008 protocol[39].

For the orthotopic models (n = 3–5) PET/CT scans were performed as described above.

### Immunohistochemistry

MT1-MMP expression was analysed on a human glioma tissue microarray (TMA) from the CNIO Biobank, comprised by a total of 40 different formalin-fixed paraffin-embedded samples in duplicate, 20 of high grade glioma (HGG, WHO grade IV, GBM) and 20 of low grade glioma (LGG, 15 of WHO grade II and 5 of WHO grade I). Immunohistochemical staining was performed using anti-MT1-MMP LEM 2/15 antibody at 1:400 dilution after antigen retrieval with low pH buffer in Autostainer platform (Dako) and counterstained with hematoxylin. Slides were digitalized using the Mirax Scan (Carl Zeiss AG, Oberkochen, Germany) and pictures were taken with the Pannoramic Viewer software (3D Histech Ltd., Ramsey, NJ, USA).

On the other hand, tumor-bearing mice were sacrificed after the last 48 h PET imaging and tumors or brains were excised, fixed in 10% buffered formalin (Sigma) and embedded in paraffin. For histopathological analysis, tissues were serially sectioned (3 μm) and stained with hematoxylin and eosin (H&E). For analysis of MT1-MMP expression, immunohistochemical staining was performed as described above.

For double staining immunohistochemistry, slides were incubated sequentially with the appropriate first and second primary antibodies as detailed: anti-MT1-MMP LEM 2/15 antibody (1/400) followed by rabbit polyclonal anti-CD31 (1:50, Abcam ab28364) or anti-MT1-MMP LEM 2/15 antibody (1/400) followed by pre-diluted rabbit monoclonal anti-Ki67 (clone MIB1, Dako). After the primary antibody, slides were incubated with the corresponding visualization systems as needed (OmniRabbit, Ventana; Roche or Goat anti Rabbit; Dako) conjugated with horseradish peroxidase and UltraMap anti-Rb Alk Phos (Ventana; Roche) conjugated with alkaline phosphatase. Immunohistochemical reaction was developed using using 3,30-diaminobenzidine tetrahydrochloride (DAB) as chromogen (Chromomap DAB; Ventana, Roche) or naphthol-AS-phosphate and fast red (Chromomap Red; Ventana, Roche). Finally, nuclei were counterstained with Carazzi’s hematoxylin. Digital slides were acquired as described above.

### Evaluation of the BBB disruption by Evans Blue staining

The alteration of the BBB was determined by following procedures previously described by Manaenko et al. [40]. 6–8 weeks old athymic/nude mice were intracraneally injected with U251-TGL and TS543-TGL cells using a fixed stereotactic apparatus (Stoelting) and monitored for brain tumor development as described above for PET imaging. Littermates were mock injected with 1.5μl of PBS as controls and sacrificed at the same time. Non injected athymic/nude littermates were included as intact brain controls.

To evaluate the BBB status, mice were injected IP with 800 ul of 2% Evan's blue dye (Sigma). One hour after injection, mice were anesthetized and perfused with acidified fixative (1% PFA in 0.05 mM citrate buffer, pH 3.5). Thirty mg of brain tissue were incubated in 500 μl formamide (Sigma) to extract Evan's blue at 60°C overnight. Absorbance was measured at 610 nm and 740 nm on a plate reader (Sinergy 4, BioTek Instruments, USA).

### Ex vivo Autoradiography

After PET/CT scans at 48 hours after injection, mice were euthanized, and tumors or whole brains bearing orthotopic tumors were removed and frozen in the OCT compound (Tissue-Tek, USA). The frozen tissues were sectioned into 30-μm thick slices by a cryostat (CM 1850, Leica). Autoradiography (ARG) was acquired by exposing the frozen sections to a phosphor screen, using a STORM 820 Phosphor Imager (GE Healthcare). For staining techniques after autoradiography, frozen samples were serially cut at 10 μm and mounted on glass slides, thawed and allowed to dry for 30 minutes. Slides were then fixed in 10% neutral buffered formalin for 10 minutes. Consecutive sections were stained with hematoxylin and eosin (H&E) and for immunohistochemistry analysis with LEM2/15, as described above.

### REMBRANDT Dataset

Glioma expression data of the REMBRANDT dataset [41] were analyzed through the GlioVis portal (http://gliovis.bioinfo.cnio.es). Data can be accessed at the GlioVis portal or at The Georgetown Database of Cancer Plus (https://gdoc.georgetown.edu/gdoc/).

### Statistical Analysis

Tukey's honest significant differences (HSD) in conjunction with ANOVA were performed for multiple comparison analysis using the R statistical software. Data in bar graphs are presented as the mean±SD. Statistical analysis was performed using the Mann-Whitney test for nonparametric data and the unpaired t test for parametric data. The Grubbs test was applied to detect eventual outliers in data sets with the online “Outlier Calculator” tool (http://www.graphpad.com/quickcalcs/Grubbs1.cfm). A *P* value of 0.05 or less was considered significant.

## Results

### Validation of MT1-MMP as suitable biomarker of GBM

**Fig 1:**
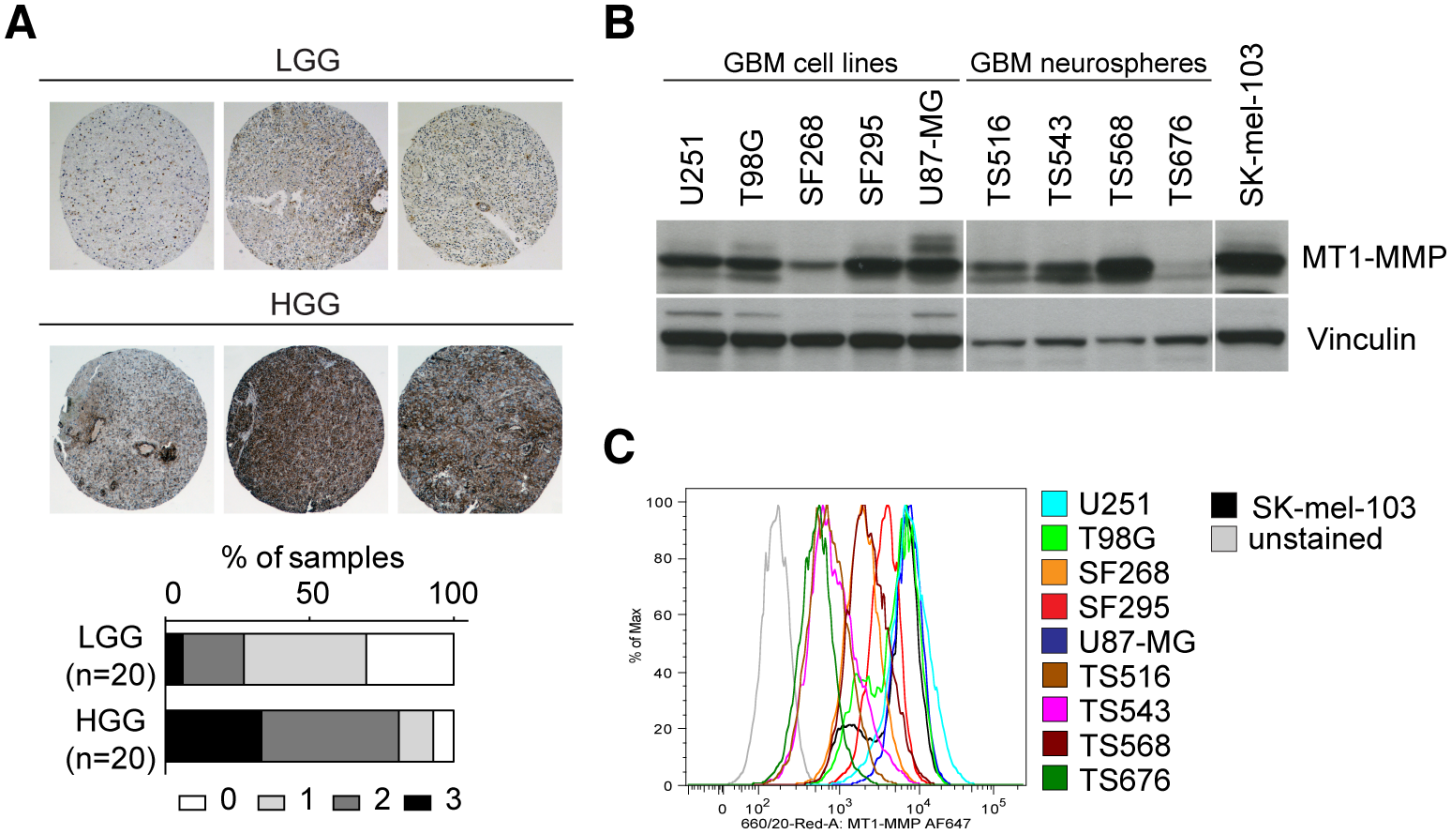
MT1-MMP protein expression analysis. **A)** Immunohistochemistry with the LEM2/15 anti MT1-MMP antibody in a TMA (n = 40) of Low-grade GBMs (LGG) and High-grade GBMs (HGG) patient tissues (representative images). Right panel: MT1-MMP staining intensity score (0–3). **B)** Immunoblotting and **C)** FACS analyses of the indicated cell lines using the LEM2/15 anti MT1-MMP antibody.

To determine whether MT1-MMP could be considered as an appropriate biomarker for gliomas, we studied its expression in a panel of 40 human glioma samples (WHO grade I, II and IV) by tissue microarray immunohistochemical analysis, using the anti-MT1-MMP LEM 2/15 antibody. A higher level of expression of MT1-MMP in GBM (HGG) was found as compared to low-grade gliomas (LGG) (Fig 1A) and it could be detected mainly in tumors but also in some endothelial cells. Moreover, analysis of a panel of different glioma cell lines and tumor neurospheres (TS) by immunoblot (Fig 1B) and FACS (Fig 1C) showed high MT1-MMP expression. Importantly, the ability of LEM-2/15 mAb to recognize intact MT1-MMP by FACS highlights its potential use for in vivo applications like immunoPET. Further validation with glioma patient public datasets was performed confirming that an increase of MT1-MMP expression at the mRNA level in GBM is observed compared to LGGs as oligodendroglioma and astrocytoma (grade II and III) (S1A and S1B Fig) and that high levels of MT1-MMP are associated with worse prognosis and a decreased survival in GBM patients (S1C Fig).

### Characterization and stability of ^89^Zr-DFO-LEM2/15

Selected conjugation conditions comprised the addition of a five-fold molar excess of DFO-NCS to the LEM2/15 solution, a reaction pH of 9.0, and incubation for 40 min at 37°C. These conditions resulted in a chelate:mAb substitution ratio of about 2–3:1. The radiochemical yield, purity, and specific activity of the ^89^Zr-DFO-LEM 2/15 used in this study were >90%, >98% and 78.5 ± 7.2 MBq/mg (n = 4) respectively, assuming virtually complete recovery of the DFO-mAb conjugates after size exclusion chromatography. Incubation of ^89^Zr-DFO-LEM 2/15 in human serum for 7 days, at 4°C or 37°C, revealed a stability of 99.2 ± 0.5% (n = 3) and 99.1± 0.3% (n = 3), respectively.

### Biodistribution and pharmacokinetics of ^89^Zr-LEM 2/15

**Fig 2:**
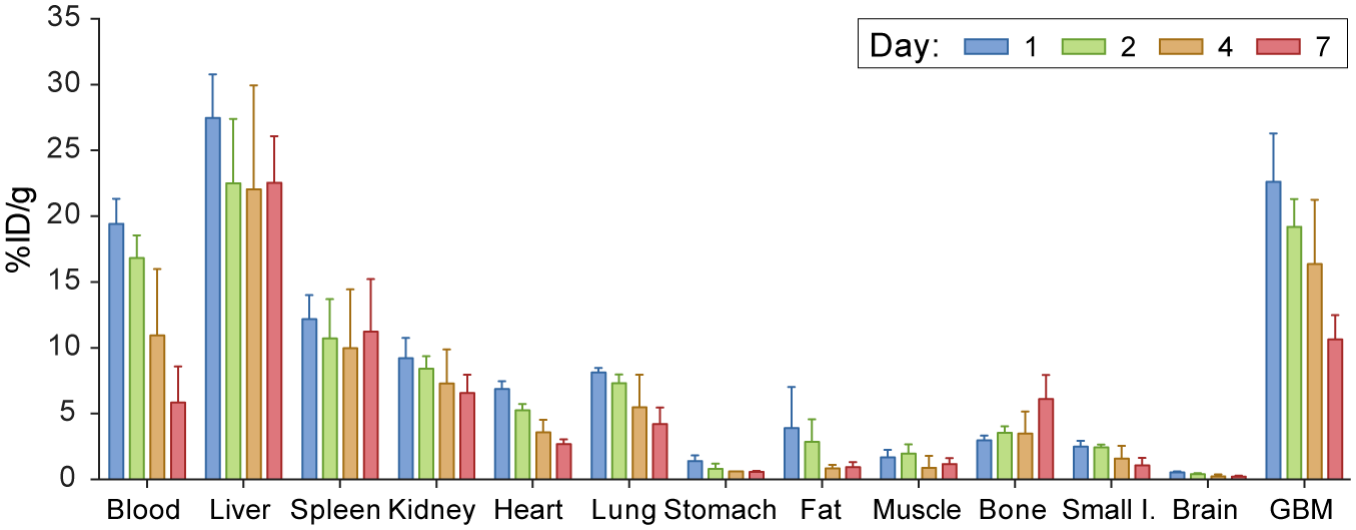
Biodistribution of ^89^Zr-DFO-LEM2/15 in mice bearing MT1-MMP^+^ GBM cells (U251).

Biodistribution experiments using ^89^Zr-LEM 2/15 (Fig 2) were conducted in nude mice bearing MT1-MMP expressing U251 and revealed a normal antibody organ distribution. In GBM tumors, the higher uptake is observed 24h post-injection (22.63±3.67%ID/g) and then it decreased in a time-dependent manner. Data showed high liver retention (27.47±3.3%ID/g)) compared to the spleen, kidneys and lungs, spanning a range between 5–15%ID/g which is representative of the hepatic clearance of full-length IgGs. The activity of the rest of organs and tissues was low (often below 5%ID/g) and decreased gradually with time except for bone which shown an increasing uptake along time.

Pharmacokinetic studies determined that the terminal phase half-life and the mean residence times were 3.2 and 4.5 days, respectively, and the volume of distribution at steady state was 2.15 mL (84 mL/kg). The systemic clearance was 0.47 mL/d (18.4 mL/d/kg), value in agreement with those obtained for therapeutic monoclonal antibodies in mice[42].

### ^89^Zr-DFO-LEM 2/15 PET imaging of subcutaneous glioma xenografts

**Fig 3:**
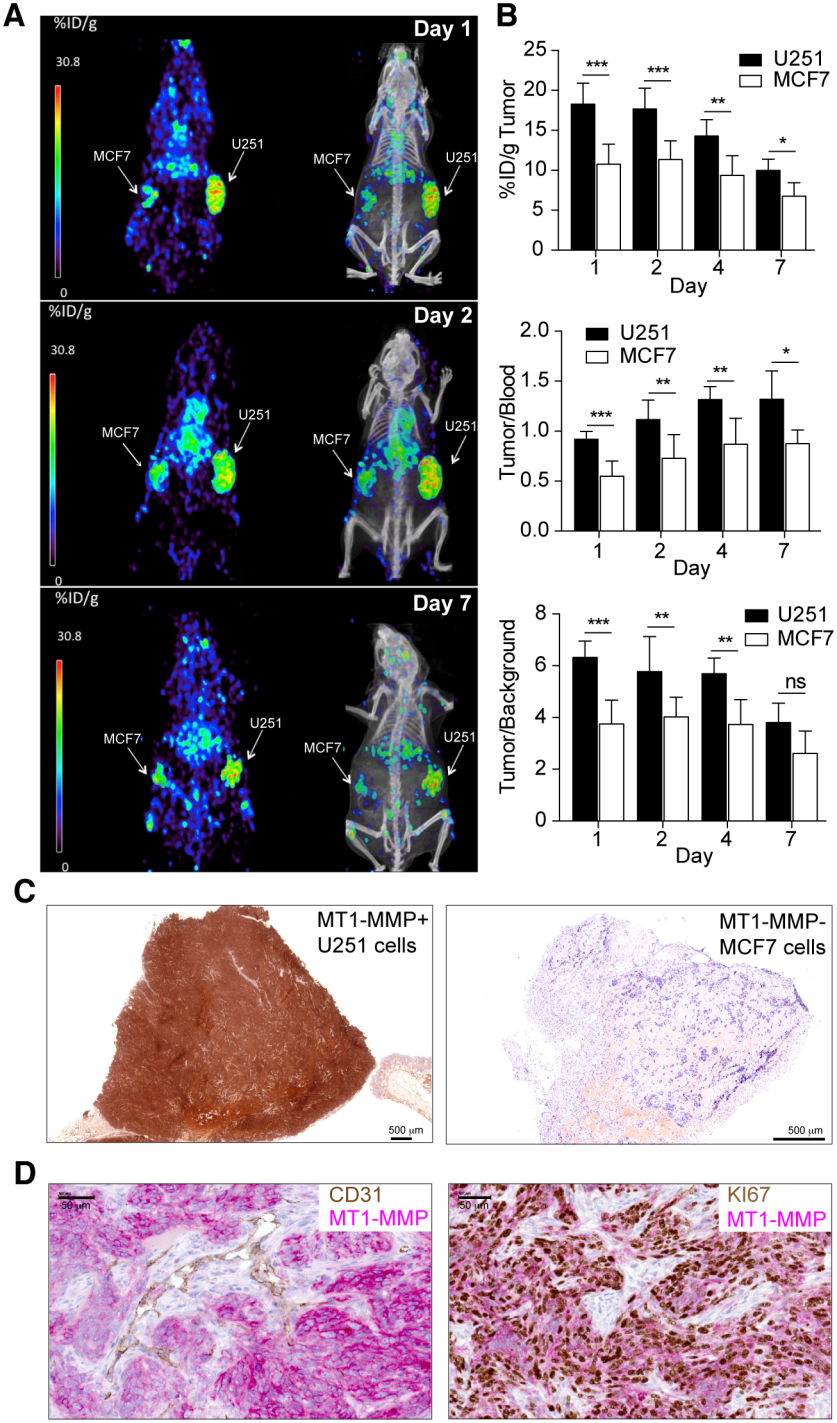
PET/CT imaging with radiolabeled ^89^Zr-DFO-LEM 2/15 in mice bearing MT1-MMP+ GBM cells (U251) and MT1-MMP- breast cancer cells (MCF-7). **A)** Representative coronal whole-body PET and CT sections at 1, 2 and 7 days p.i.; **B)** Levels of radioactivity in tumors, tumor to blood and tumor to background ratios derived from PET imaging after ^89^Zr-DFO-LEM 2/15 administration to mice bearing MT1-MMP+ and MT1-MMP- tumors (mean±SD, n = 11−4/time); **C)** Immunohistochemistry of tumor tissue from xenografted mice used for PET imaging. MT1-MMP was detected using LEM2/15 antibody. Scale bars: 500 μm **D)** Representative images of double immunostaining for MT1-MMP (pink) and CD31 vascular marker (brown) (left panel) and for MT1-MMP (pink) and Ki67 proliferation marker (brown)(right panel) in U251 tumor implants from xenografted mice used for PET imaging. Scale bars: 50 μm. Significant differences: p<0.05 (*), p<0.001 (**) and p<0.0001 (***), ns, not significant.

^89^Zr-DFO-LEM 2/15 small-animal PET was conducted on mice harboring xenograft tumors at opposite flanks with MT1-MMP^+^ GBM U251 cells and MT1-MMP^-^ breast MCF7 cells. Assessment of MT1-MMP expression in U251 and MCF7 by FACS and immunoblotting confirmed high and null expression of U251 and MCF7, respectively (data not shown). Levels of radioactivity in tumors, tumor-to-blood and tumor-to-background ratios were calculated. In tumors, radioactivity decreased in a time-dependent manner, with the uptake being 18.3±2.6 (n = 11), 17.7±2.6 (n = 8), 14.3±2.0 (n = 6) and 10±1.4 (n = 4) %ID/g at 1, 2, 4 and 7 days p.i., respectively in MT1-MMP^+^ U251 tumors, and 10.8±2.5 (n = 11), 11.4±2.3 (n = 8), 9.4±2.4 (n = 6) and 6.8±1.7 (n = 4) %ID/g at 1, 2, 4 and 7 days p.i., respectively in MT1-MMP^-^ MCF-7 tumors (Fig 3A and 3B top panel and S1 Video). The uptake in MT1-MMP^+^ tumors was significantly higher than in MT1-MMP^-^ tumors at all times. Tumor/blood ratios in GBM/MT1-MMP^+^ tumors were 0.88±0.08, 1.07±0.19, 1.29±0.12 and 1.31±0.29 at 1, 2, 4 and 7 days post-injection, respectively (Fig 3B middle panel). However, tumor/blood ratios in breast MT1-MMP^-^ cells were significantly lower (0.51±0.15, 0.67±0.23, 0.81±0.24 and 0.80±0.11 at 1, 2, 4 and 7 days post-injection, respectively) than those for GBM/MT1-MMP^+^ tumors. Tumor/background ratios in GBM/MT1-MMP^+^ tumors exhibited high values after 1 day (6.32±0.62, 5.77±0.77, 5.68±0.61 and 3.80±0.74 at 1, 2, 4 and 7 days p.i., respectively) in contrast to significantly lower values obtained with breast MT1-MMP^-^ tumors (3.74±0.92, 4.02±0.75, 3.73±0.95 and 2.60±0.86 at 1, 2, 4 and 7 days p.i., respectively) (Fig 3B bottom panel). In addition, immunohistochemical staining of tumors excised 48h after PET scan by using the LEM2/15 antibody confirmed high expression of MT1-MMP in the U251 xenograft (Fig 3C, left panel) and its absence in the MCF7 tumor (Fig 3C, right panel). To obtain more detailed information with regard to the localization of MT1-MMP expression within the tumor, double staining immunohistochemistry analyses with LEM2/15 antibody and tumor (Ki67) or vascular (CD31) markers were performed (Fig 3D). We determined that tumor cells (Ki67+) immunostained strongly positive for MT1-MMP while most endothelial cells (CD31+) did not stain at all. This would suggest that LEM2/15 does not recognize properly mouse MT1-MMP, therefore we could exclude that the PET signal come from the endothelial cells. Digital autoradiography and histologic staining revealed a homogeneous distribution of ^89^Zr-DFO-LEM 2/15 in the U251 tumors corresponding to the MT1-MMP-expressing regions (S2A Fig).

### ^89^Zr-DFO-LEM 2/15 PET imaging of intracranial xenograft tumors

**Fig 4:**
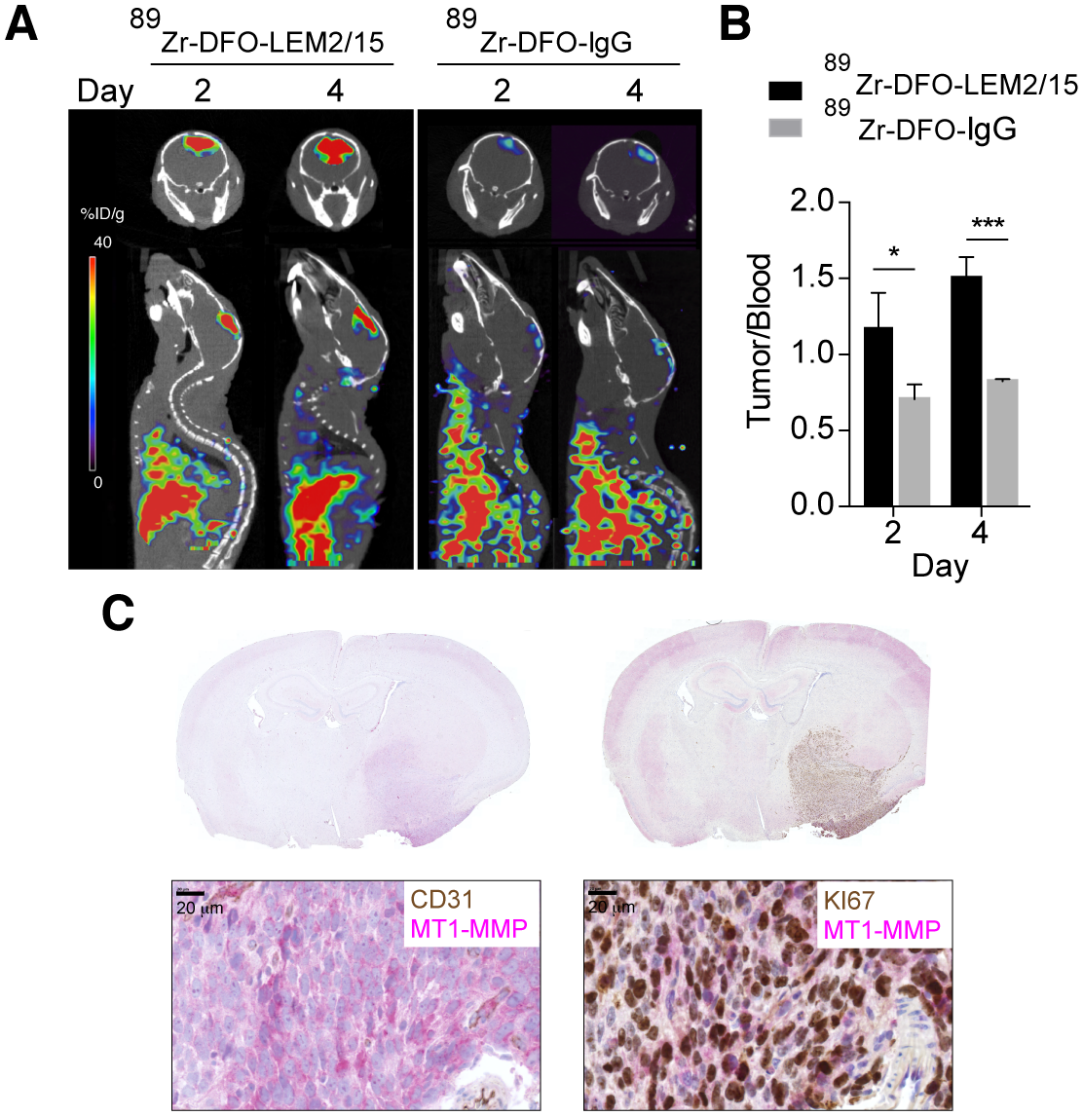
PET/CT imaging with radiolabeled ^89^Zr-DFO-LEM2/15 in mice bearing orthotopic xenografts containing patient-derived neurospheres. **A)** Representative fused PET/CT images of coronal and sagittal planes at 2 and 4 days p.i. containing TS543 brain tumors with ^89^Zr-DFO-LEM2/15 (left panels) and ^89^Zr-DFO-IgG1 as isotype control (right panels) **B)** Tumor-to-blood ratios for ^89^Zr-DFO-LEM2/15 (black bars) and ^89^Zr-DFO-IgG1 (grey bars) in nude mice bearing orthotopic TS453 xenografts at 2 and 4 days p.i. derived from PET imaging. Values are presented as mean±SD (n = 3–5). **C)** Representative brain images of double immunostaining for MT1-MMP (pink) and CD31 vascular marker (brown) (left panel) and for MT1-MMP (pink) and Ki67 proliferation marker (brown) (right panel) from orthotopic TS453 xenografts used for PET imaging. Higher-magnification are shown in the bottom panels. Scale bars: 20 μm. Significant differences: p<0.05 (*) and p<0.0001 (***).

To determine the applicability of PET imaging to GBM tumor in a more clinically relevant mouse orthotopic model, PET/CT imaging with ^89^Zr-DFO-LEM2/15 and a control ^89^Zr-DFO-IgG1 was performed 10 days after intracranial injections of patient-derived neurospheres (TS543). Presence of tumors was confirmed by bioluminiscence. The brain PET image (Fig 4A and S2 Video) shows that ^89^Zr-DFO-LEM2/15 was specifically accumulated in tumor, enabling its accurate localization and delineation. Posterior quantitative analysis of tumor/blood ratios of ^89^Zr-DFO-LEM2/15 compared with control ^89^Zr-DFO-IgG1 distribution (1.17±0.23 and 1.50±0.14 versus 0.71±0.10 and 0.82±0.01 at 2 and 4 days p.i., respectively) revealed significant differences over time (Fig 4B and S3A Fig), indicating an MT1-MMP-mediated specific tumor uptake in this model. Sections of mouse brains bearing the GBM xenografts immunostained for MT1-MMP confirmed the expression of this protein in the tumor tissue but not in the surrounding brain tissue or in vascular endothelial cells, where most of the tumor vessels were negative for LEM2/15 staining (Fig 4C).

Interestingly, the ^89^Zr-DFO-LEM2/15 antibody only caused a very weak signal in an orthotopic U251 tumor model and did not show a significant difference between tumor/blood ratios of the specific labeled antibody and control ^89^Zr-DFO-IgG1 (0.23±0.04 and 0.30±0.03 versus 0.17± 0.05 and 0.21± 0.10, at 2 and 4 days p.i., respectively) (S3A Fig), despite the considerably higher expression of MT1-MMP on U251 cells as compared with TS543 neurospheres (Fig 1C). To test whether the differences in the ^89^Zr-DFO-LEM2/15 uptake, between the U251 and TS543 orthotopic models, were associated to the integrity of the blood-brain barrier (BBB) we performed an Evans blue (EB) staining. U251 tumors showed significantly lower EB staining than TS543 tumors (9.14±3.14 versus 39.35±26.83 μg of EB/g tissue, respectively; intact or mock surgery brain: 2.12±0.77 or 2.03±0.51μg of EB/g tissue, respectively) (S3B Fig), indicating only partially increased BBB-permeability in the U251 model and a highly disrupted BBB in the TS543 model.

In conclusion, small animal PET/CT using ^89^Zr-DFO-LEM2/15 mAb enabled the specific and efficient detection of aggressive TS543 neurosphere-derived tumors in which the BBB is disrupted.

## Discussion

GBM remains one of the most deadly form of human cancer despite tremendous advances in surgical approaches, radiotherapy and chemotherapy over the last decades, with an average survival of 14–16 months using the current standard of care based on administration of radiotherapy and concomitant and adjuvant temozolomide [43][44]. Therefore, significant improvements in understanding of molecular pathogenesis, as well as in diagnosis and novel therapeutic strategies should be made to increase patient survival rates [45]. In particular, accurate diagnosis has a key role in clinical management of GBM patients. Treatment follow-up and clinical decision-making are currently based on the accuracy of non-invasive imaging techniques such as MRI or PET targeting aberrant metabolic processes. MRI has limitations in identifying tumor grade, invasive growth into neighboring tissue, treatment-induced changes and recurrences because pseudoprogression and tumor progression cannot be properly distinguished [46]. PET imaging with ^18^F-FDG and especially other PET tracers such as ^11^C-MET, ^11^C-TYR, ^18^F-FET or ^18^F-DOPA have shown a great potential for cerebral GBM diagnosis, however their sensitivity and specificity for detecting tumor growth or shrinkage remains to be fully validated [47]. These drawbacks make necessary to seek urgently other noninvasive biologic-based imaging with molecularly targeted agents such as immunoPET [48] that might improve the specificity of tumor diagnosis compared with the more anatomical based techniques.

Herein, we provide proof of concept that MT1-MMP expression can be quantitatively assessed by an immunoPET using a specific monoclonal antibody (LEM 2/15), readily labeled with ^89^Zr and used for *in vivo* tumor imaging. Furthermore, we evaluated the *in vitro* and *in vivo* properties of this labeled antibody to develop a new PET probe for glioma detection, showing that ^89^Zr-DFO-LEM2/15-mediated PET yields high resolution detailed images with high tumor-to-background contrast in flank and orthotopic xenograft models of GBM. Therefore, this study was performed to establish and validate MT1-MMP as a candidate for GBM diagnosis by means of noninvasive PET imaging.

We chose the function-blocking antibody LEM2/15 because it shows very high specificity for MT1-MMP. It was generated against the V-B loop of MT1-MMP (residues 218–233) [27] which is located outside the conserved catalytic cleft and therefore its epitope displays low sequence homology among the MMP family. Recently, the crystal structure of Fab LEM2/15 complexed with the catalytic domain of MT1-MMP has shed light on the molecular mechanism of the allosteric inhibition of this enzyme by the antibody [28]. This work pointed to the potential utility of LEM2/15 as lead for the development of novel MMP-targeted therapeutics [29]. In this context, our goal would also be the integration of LEM2/15-based PET into the process of the drug development in preclinical studies and eventually for clinical translation to monitor noninvasively target expression.

First, we tested whether MT1-MMP was indeed a suitable target for a PET biomarker of GBM by studying extensively MT1-MMP expression at different levels. Utilizing bioinformatics analysis of the REMBRANDT dataset, we confirmed a significant correlation between increased MT1-MMP expression with advanced tumor grades, malignancy and shorter overall survival for GBM patients, in concordance with previously reported results[15,17,49]. Further validation was carried out at protein level, detecting MT1-MMP in a panel of glioma cells by immunoblotting and FACS; and in a TMA immunohistochemistry staining with glioma samples of various grades, in which we documented a marked increase in expression of MT1-MMP in GBM compared to low-grade gliomas. Altogether, our data suggested the feasibility of MT1-MMP to become a suitable molecular target for GBM diagnosis. MT1-MMP overexpression would indicate tumor progression towards more aggressive phenotypes associated with a worse prognosis, and also predict that the LEM2/15 antibody would have potential utility as MT1-MMP-targeted PET probe for imaging of GBM.

Previous studies have explored the use of MT1-MMP as imaging biomarker in SPECT[31,32,50], optical imaging[30] and PET/optical imaging using cleavable PEGylated MT1-MMP substrate peptide probes conjugated with ^18^F-labeled BODIPY (boron dipyrromethene) scaffold which allows an effective integration of fluorescence-based imaging with PET studies to monitor MT1-MMP proteolytic activity [51]. However, to the best of our knowledge, this is the first time that a MT1-MMP-based immunoPET is described for GBM detection and it is expected to have greater performance than other MT1-MMP-based imaging systems developed up to now: immunoPET combines the antibody characteristics of high specificity, affinity and great structural flexibility to generate a variety of fragments with half-lives matching optimally the period of semi-desintegration of isotopes, with PET as imaging technique which provides higher spatial resolution and sensitivity than SPECT from a clinical perspective.

Concerning radiolabeling, experiments on different Zr coordination methods have reported that ^89^Zr-DFO mAbs provides higher *in vivo* stability with respect to demetalation and relatively low levels of radiotracer accumulation in background tissue, especially in bones[52] and hence DFO is currently the best chelator for ^89^Zr[53]. The results reported here are consistent with previous investigations and confirmed that ^89^Zr is a suitable radionuclide for labeling intact antibodies[33,54,55]. Biodistribution studies with the U251 murine model revealed specific MT1-MMP driven uptake of ^89^Zr-DFO LEM2/15 in GBM tumors (about 30%ID/g at 24h), declining in a time-dependent manner up to 15% ID/g at 7 days p.i. Most likely this increased clearance of radioactivity at tumor site represents metabolism instead of loss of ^89^Zr, due to lack of complex stability since ^89^Zr-DFO-LEM2/15 was very stable in serum, with no significant dissociation of the complexes after 7 days. In non-target tissues, organs of the reticuloendothelial system showed relatively high uptake, especially liver, which is attributable to the biological elimination of the tracer, given that antibodies are usually cleared through their interaction with the Fc receptors expressed on cells of this system[56], and is a pattern frequently observed with radiolabeled antibodies[57–59]. Also, an increasing accumulation of activity in the bones is observed along time, just the same as in previous studies using DFO as a chelator [60], since Zr ion shows a high affinity for bones[52].

Regardless of the antibody/antigen system, no *in vivo* investigation of an ^89^Zr-DFO-labeled imaging probe is complete without a demonstration of selectivity. This can be achieved using a cell line that does not express the antigen in question (i.e., MT1-MMP), such as MCF-7 cell line used in the present study. To confirm that the accumulation of ^89^Zr-DFO-LEM2/15 was attributable to specific rather than non-specific bindings, we implanted MT1-MMP-expressing and non-expressing tumor cells within the same animal in the microPET/CT experiments. Tumor/blood ratios of ^89^Zr-DFO LEM2/15 in GBM MT1-MMP^+^ tumors were significantly higher than those in breast MT1-MMP^-^ tumors, indicating the specificity of ^89^Zr-DFO-LEM2/15 toward MT1-MMP. The unspecific accumulation of the antibody in breast tumors could be due to the enhanced permeability and retention (EPR) effect, which is characterized by a leaky nature of tumor vasculature and reduced lymphatic drainage [61], rather than an involvement of the vasculature since LEM2/15 showed no reactivity with most of the mouse tumor blood vessels by IHC. In fact, it has been previously described that LEM2/15 antibody reacted poorly with MT1-MMP from murine origin [62].

Compared to some limitations of the subcutaneous model, orthotopic tumor models more closely mimic the natural biologic behavior and characteristics of human tumors because they grow in its native microenvironment and reliably replicate certain important characteristics. For the generation of orthotopic GBM xenografts we employed neurospheres in addition to established glioma cell lines such as U251 because these grafted masses structurally resemble more to the tumor of origin. Intact antibodies are too large to cross readily the BBB[63] and indeed we observed that a disrupted BBB is needed for the LEM2/15 antibody to penetrate into the brain tumors. Accordingly, ^89^Zr-DFO LEM2/15 was able to detect orthotopically growing GBM implants from TS543 but not from U251, which correlates with the integrity of the BBB, as analyzed by Evans blue staining. More brain tumor models with different BBB leakage will be utilized in the future to test the tracer activity. However, this high dependence on the BBB integrity for targeting is a major limit for the use of intact LEM2/15 in clinical developments.

To overcome such a limitation, our current work is aimed at extending this initial findings using engineered and miniaturized derivatives of the LEM2/15 antibody. The minituarized antibodies can enhance BBB penetration and therefore result in an improved tumor-targeted imaging[64]. Moreover labelling with PET isotopes characterized by short or intermediate half-lives such as ^68^Ga[65] would optimize the pharmacokinetics and facilitate wide access to this technology since ^68^Ga can potentially be generator-produced on site (rather than cyclotron produced). Besides, future studies with different tumor grades are needed to confirm the correlation between the MT1-MMP-targeted PET and the different tumor grades. Also, the demonstration of the feasibility of MT1-MMP as biomarker for tumor progression, able to discriminate between real and pseudoprogression after standard treatments, would be of great relevance for evaluating MT1-MMP-specific antibodies as non-invasive diagnostic tools in clinic.

In conclusion, we established the feasibility of the use of MT1-MMP-based immunoPET for an efficient detection of GBM and it opens the possibility of using engineered anti-MT1-MMP mAbs to more accurate biological characterization and diagnosis of GBM, as an alternate to non-specific tracers.

## Supporting Information

S1 Fig MT1-MMP expression in GBM patients. **A)** MT1-MMP mRNA expression in the REMBRANDT dataset stratified by histology: Non-tumor (n = 28), Oligodendroglioma (n = 67), Astrocytoma (n = 147) and GBM (n = 219). Right panel: results of a Tukey’s HSD post hoc test showing the differences between mean levels for each comparison and the 95% confidence interval. **B)** Same as in A) with the patients stratified by tumor grade: Grade II (n = 98), Grade III (n = 85), Grade IV (n = 130). **C)** Kaplan Meier survival estimates of GBM patients in the REMBRANDT dataset stratified by the median MT1-MMP mRNA expression.
(TIF)

S2 Fig **(A)** Hematoxylin and eosin (Left), immunohistochemistry with LEM2/15 antibody (Center) and ex vivo autoradiography (Right) of the U251 s.c. xenograft from mouse injected with ^89^Zr-DFO-LEM2/15, indicating there was specific uptake in the U251 tumor. **(B)** Hematoxylin and eosin (Left), immunohistochemistry with LEM2/15 antibody (Center), and ex vivo autoradiography (Right) of the brain from a mouse with a TS543 orthotopic xenograft injected with ^89^Zr-DFO-LEM2/15, confirming colocalization of the tracer with MT1-MMP expression.
(TIF)

S3 Fig **(A)** Uptake of ^89^Zr-DFO-LEM2/15 and ^89^Zr-DFO-IgG1 as isotype control in orthotopic TS543 and U251 xenograft models, represented as %ID/g tumor (left panel) and tumor-to-blood ratio (right panel) at days 2 and 4 p.i. Data of tumor-to-blood ratios from TS543 tumors are also shown in Fig 4, they are included here just for comparison. Horizontal bars indicate medians. Red and blue lines represent ±SDs in TS543 and U251 xenografts, respectively, (n = 3–5). **(B)** Analysis of BBB integrity by quantification of intravenously administered Evans blue in the brain of mice with intact (●), mock surgery brains (■), orthotopic U251 (▲) and TS543 (▼) xenograft models. Horizontal bars indicate medians and vertical bars, ±SDs (n = 3).
(TIF)

S1 Video PET-CT 3D render of a representative mouse bearing MT1-MMP+ GBM cells (U251), right flank, and MT1-MMP- breast cancer cells (MCF-7), left flank, labeled with ^89^Zr-DFO-LEM 2/15 at 24 h post injection. (MPG)

S2 Video PET/CT 3D render imaging of a representative mouse bearing orthotopic xenograft containing patient-derived TS543 neurospheres and labeled with ^89^Zr-DFO-LEM 2/15 at 24h after injection (MPG)
