# Supplementary figures and images for: Targeting MT1-MMP as an ImmunoPET-Based Strategy for Imaging Gliomas
Source: PLoS One. 2016 Jul 27;11(7):e0158634. doi: 10.1371/journal.pone.0158634 (PMC4962974; doi:10.1371/journal.pone.0158634)

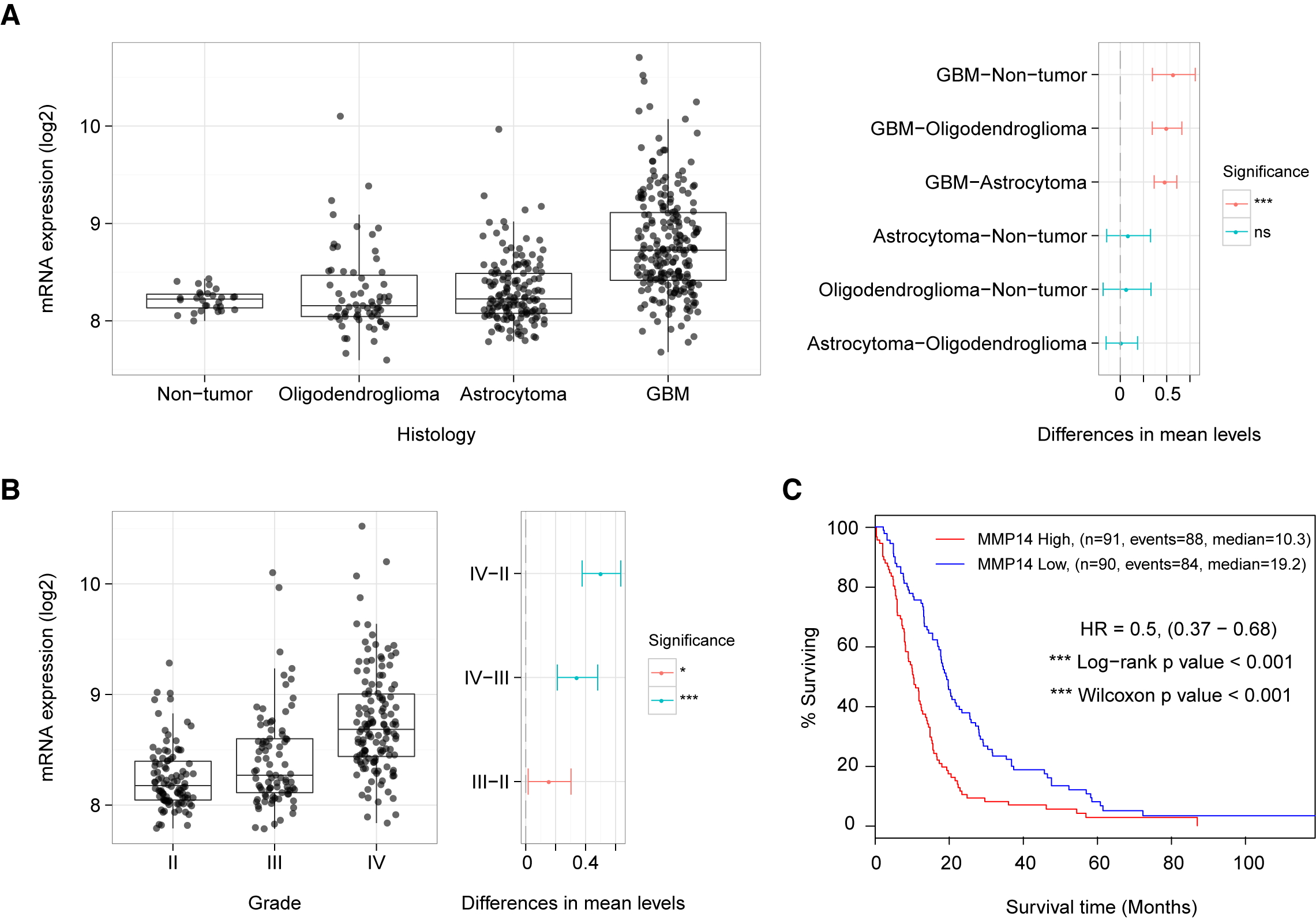

Supplement: S1 Fig — A) MT1-MMP mRNA expression in the REMBRANDT dataset stratified by histology: Non-tumor (n = 28), Oligodendroglioma (n = 67), Astrocytoma (n = 147) and GBM (n = 219). Right panel: results of a Tukey’s HSD post hoc test showing the differences between mean levels for each comparison and the 95% confidence interval. B) Same as in A) with the patients stratified by tumor grade: Grade II (n = 98), Grade III (n = 85), Grade IV (n = 130). C) Kaplan Meier survival estimates of GBM patients in the REMBRANDT dataset stratified by the median MT1-MMP mRNA expression. (TIF) [file pone.0158634.s001.tif]

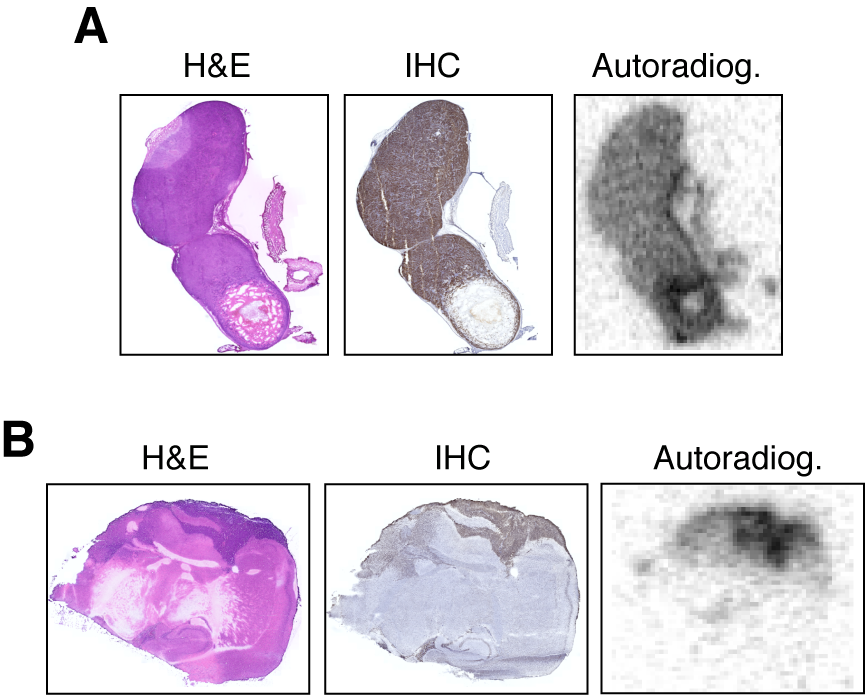

Supplement: S2 Fig — (A) Hematoxylin and eosin (Left), immunohistochemistry with LEM2/15 antibody (Center) and ex vivo autoradiography (Right) of the U251 s.c. xenograft from mouse injected with 89Zr-DFO-LEM2/15, indicating there was specific uptake in the U251 tumor. (B) Hematoxylin and eosin (Left), immunohistochemistry with LEM2/15 antibody (Center), and ex vivo autoradiography (Right) of the brain from a mouse with a TS543 orthotopic xenograft injected with 89Zr-DFO-LEM2/15, confirming colocalization of the tracer with MT1-MMP expression. (TIF) [file pone.0158634.s002.tif]

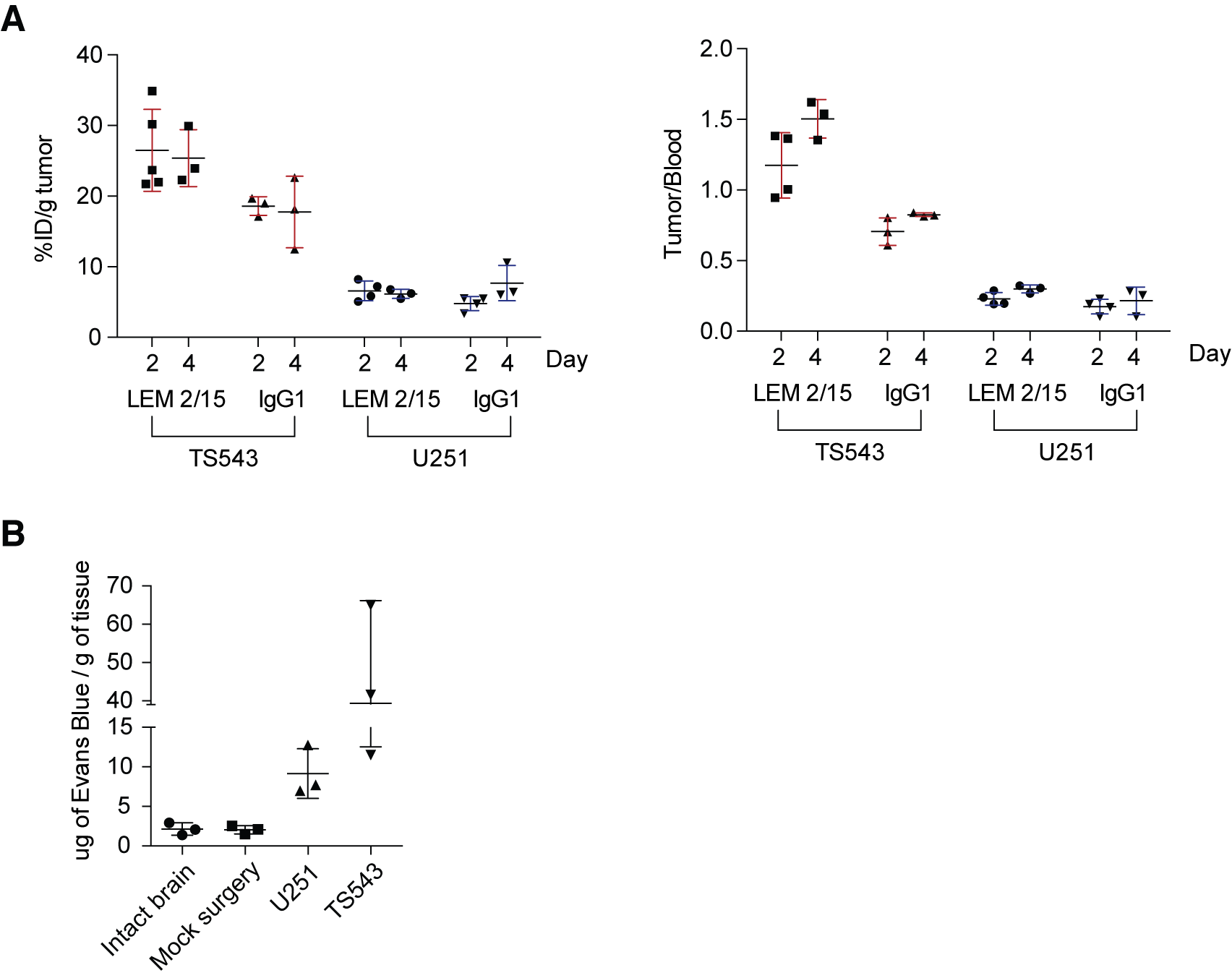

Supplement: S3 Fig — (A) Uptake of 89Zr-DFO-LEM2/15 and 89Zr-DFO-IgG1 as isotype control in orthotopic TS543 and U251 xenograft models, represented as %ID/g tumor (left panel) and tumor-to-blood ratio (right panel) at days 2 and 4 p.i. Data of tumor-to-blood ratios from TS543 tumors are also shown in Fig 4, they are included here just for comparison. Horizontal bars indicate medians. Red and blue lines represent ±SDs in TS543 and U251 xenografts, respectively, (n = 3–5). (B) Analysis of BBB integrity by quantification of intravenously administered Evans blue in the brain of mice with intact (●), mock surgery brains (■), orthotopic U251 (▲) and TS543 (▼) xenograft models. Horizontal bars indicate medians and vertical bars, ±SDs (n = 3). (TIF) [file pone.0158634.s003.tif]
